# Cytokine Thresholds in Gingival Crevicular Fluid with Potential Diagnosis of Chronic Periodontitis Differentiating by Smoking Status

**DOI:** 10.1038/s41598-018-35920-4

**Published:** 2018-12-20

**Authors:** N. Arias-Bujanda, A. Regueira-Iglesias, M. Alonso-Sampedro, M. M. González-Peteiro, A. Mira, C. Balsa-Castro, I. Tomás

**Affiliations:** 1 Oral Sciences Research Group, Special Needs Unit, Department of Surgery and Medical-Surgical Specialties, School of Medicine and Dentistry, Health Research Institute Foundation of Santiago (FIDIS), Universidade de Santiago de Compostela, Galicia, Spain; 2 Department of Internal Medicine and Clinical Epidemiology, Complejo Hospitalario Universitario, Santiago de Compostela, Galicia, Spain; 3 Center for Advanced Research in Public Health, FISABIO Foundation, Valencia, Spain

## Abstract

The objective of the present study was to determine cytokine thresholds derived from predictive models for the diagnosis of chronic periodontitis, differentiating by smoking status. Seventy-five periodontally healthy controls and 75 subjects affected by chronic periodontitis were recruited. Sixteen mediators were measured in gingival crevicular fluid (GCF) using multiplexed bead immunoassays. The models were obtained using binary logistic regression, distinguishing between non-smokers and smokers. The area under the curve (AUC) and numerous classification measures were obtained. Model curves were constructed graphically and the cytokine thresholds calculated for the values of maximum accuracy (ACC). There were three cytokine-based models and three cytokine ratio-based models, which presented with a bias-corrected AUC > 0.91 and > 0.83, respectively. These models were (cytokine thresholds in pg/ml for the median ACC using bootstrapping for smokers and non-smokers): IL1alpha (46099 and 65644); IL1beta (4732 and 5827); IL17A (11.03 and 17.13); IL1alpha/IL2 (4210 and 7118); IL1beta/IL2 (260 and 628); and IL17A/IL2 (0.810 and 1.919). IL1alpha, IL1beta and IL17A, and their ratios with IL2, are excellent diagnostic biomarkers in GCF for distinguishing periodontitis patients from periodontally healthy individuals. Cytokine thresholds in GCF with diagnostic potential are defined, showing that smokers have lower threshold values than non-smokers.

## Introduction

Periodontitis is a public health problem, as it is highly prevalent and causes disability and social inequality^1^. In 2010, severe periodontitis was estimated to be the sixth most prevalent disease globally, affecting 743 million people worldwide^2^. Periodontitis is currently being connected bidirectionally to the pathogenesis of various conditions and systemic diseases of high morbi-mortality such as diabetes, coronary heart disease, metabolic syndrome, chronic respiratory diseases, rheumatoid arthritis, cancer, obstetric complications and cognitive impairment^3,4^.

It is now widely accepted that, although the initiating factor is a polymicrobial dysbiosis^5^, the pathogenesis of periodontitis is driven by the development of a chronic inflammatory host immune response^6,7^. The nature and extent of this response are fundamental determinants of the susceptibility to and progression of periodontitis^6,7^.

Cytokines are soluble protein ‘messenger’ molecules produced by a variety of cells that transmit signals to other cells^8^. Cytokines play a crucial role in initiating and sustaining the inflammatory immune response by stimulating the production of secondary mediators. These mediators, in turn, evoke a cascade of events that amplify the inflammatory response and induce the production of enzymes that are responsible for the degradation of connective tissue and osteoclastic bone resorption^9^.

Cytokines interact and function within a complex and dynamic network of interactions, rather than being dominated by the action of individual cytokines^8^. In fact, an imbalance between the pro-inflammatory and anti-inflammatory cytokines derived from Th1, Th2, Th17 and Treg lymphocyte subpopulations is suggested as being responsible for periodontal breakdown through cellular and humoral hyper-immune responses^10^. However, the reductionist approach is predominant *in vivo* research, as very few authors have analysed the simultaneous presence of more than 10 cytokines^11–13^, or more than four cytokine ratios, in gingival crevicular fluid (GCF) from periodontal patients^14,15^. Accordingly, more evidence is required from multiple cytokine analyses to increase what is understood of this complex and dynamic network^8^.

On the other hand, the detection of biomarkers in GCF for predicting the early onset of periodontitis or evaluating the untreated or treated disease activity is a key challenge in periodontology^16–18^. There is, however, limited literature on the development and validation of predictive models based on GCF cytokine levels for the diagnosis or prognosis of periodontitis^19,20^.

Accordingly, the objectives of this cross-sectional study were:

1. To compare the levels of 16 cytokines detected in GCF, as well as multiple cytokine ratios obtained from them, in periodontally healthy individuals and patients with chronic periodontitis.
2. To determine the diagnostic value thresholds derived from cytokine-based and cytokine ratio-based models in non-smokers and smokers, selecting those models with a high discriminatory capacity to distinguish between periodontal patients and periodontally healthy controls.
3. To validate cytokine-based and cytokine ratio-based models internally using bootstrapping techniques, describing their diagnostic thresholds, as well as apparent and corrected measures of discrimination and classification.

## Materials and Methods

### Selection of study groups

A sample of 150 eligible participants was recruited among 250 consecutive patients from the general population who were referred to the School of Medicine and Dentistry (Universidade de Santiago de Compostela, Spain) for an evaluation of their oral health status between 2013 and 2015. This sample consisted of all the cases (patients with the target condition, that is, 75 subjects affected by moderate to severe generalised chronic periodontitis -perio group-) and a random sample of the noncases (75 periodontally healthy controls -control group-). Patients were selected if they fulfilled the inclusion criteria, which are detailed in the footnote of Table 1.

**Table 1:** Age, gender, smoking habit and clinical characteristics associated with the periodontal status in the control and perio groups. Values are means (standard deviations) and number of subjects.

Clinical Parameters	Study Groups^a^		
	Control group (n = 74)	Perio group (n = 73)	P Value
**Age (years)**	45.65 (12.37)	51.12 (10.01)	0.005
**Gender**			
Male	31	31	NS
Female	43	42	
No. of teeth	26.72 (3.25)	25.55 (4.00)	NS
**Full mouth**			
BPL (%)	26.41 (18.66)	53.08 (26.77)	<0.001
BOP (%)	15.05 (6.61)	51.12 (20.07)	<0.001
PPD (mm)	2.11 (0.27)	3.49 (0.65)	<0.001
CAL (mm)	2.36 (0.46)	4.25 (1.12)	<0.001
**Sampled sites**			
BOP (%)	10.11 (10.24)	66.97 (23.93)	<0.001
PPD (mm)	2.23 (0.22)	5.65 (0.89)	<0.001
CAL (mm)	2.31 (0.27)	6.05 (1.12)	<0.001
**Smoking habit** ^b^			
Non-smokers	61	32	0.001
Smokers	13	41	
Cigarettes/day (no.)	8.08 (4.44)	15.20 (7.94)	0.001
Months of smoking (no.)	236.38 (155.91)	320.78 (109.03)	NS

BPL = bacterial plaque level; BOP = bleeding on probing; PPD = probing pocket depth; CAL = clinical attachment level; NS = not significant. In this series, the inclusion criteria applied were: 1) age 30 to 75; 2) no medical history of diabetes mellitus or hepatic or renal disease, or other serious medical conditions or transmittable diseases; 3) no history of alcohol or drug abuse; 4) no pregnancy or breastfeeding; 5) no intake of systemic antimicrobials during the previous six months; 6) no intake of anti-inflammatory medication in the previous four months; 7) no routine use of oral antiseptics; 8) no presence of implants or orthodontic appliances; 9) no previous periodontal treatment; 10) smokers who had stopped smoking less than five years before the sampling; and 11) the presence of at least 18 natural teeth.

^a^Of the 150 patients who fulfilled the inclusion criteria and had an adequate periodontal diagnosis, three were excluded for unexpected events. The control group included periodontally healthy individuals with BOP < 25%, no sites with a PPD ≥ 4 mm and no radiographic evidence of alveolar bone loss. Considering previously established criteria^21,22^, patients in the perio group were diagnosed with moderate to severe generalised chronic periodontitis. ^b^A patient was defined as a smoker if he/she was currently smoking and had been a smoker for at least eight years) and as a non-smoker if he/she had never smoked or had stopped smoking more than five years before the sampling.

One previously calibrated, experienced dentist performed all the periodontal examinations. The probing pocket depth (PPD) and clinical attachment level (CAL) were recorded on all teeth at six sites per tooth using a PCP-UNC 15 probe. Bleeding on probing (BOP) and bacterial plaque level (BPL) data were recorded for the full mouth on a binary scale (presence/absence) on six sites per tooth. Standardised radiographs of all teeth were obtained to assess the alveolar bone status.

The presence of periodontal health or moderate to severe generalised chronic periodontitis was established according to the clinical/radiographic information, applying previously published criteria^21,22^. Smoking histories were obtained using a questionnaire, with information collected on smoking status (never, past or current, the number of months of smoking and the number of cigarettes/day). All the answers were reviewed with the subject by a member of the study team.

This study was conducted according to the principles outlined in the Declaration of Helsinki (as revised in 2000) on experimentation involving human beings^23^. The TRIPOD guidelines were considered for further predictive analysis^24^.

### Gingival crevicular fluid sampling

The GCF collection took place one week after the initial examination, and the samples were obtained at the same time of day (in the afternoon, approximately 5–7 h after toothbrushing). A paper strip (Periopaper, Amityville, NY, USA) was inserted into the gingival sulcus or periodontal pocket for 30 sec, using a GCF collection protocol previously described^25^. GCF samples from the controls and periodontal patients were collected and pooled from 20 non-adjacent proximal sites. In the first case, samples were taken from subgingival sites from teeth in quadrants 1 and 3, and in the second case from sites from the most in-depth PPD in each quadrant.

Strips from each subject were inserted into labelled tubes with 300 ml of 0.01 M PBS (pH = 7.2) and a protease inhibitor (Complete Mini, protease inhibitor cocktail tablets, Roche Applied Science, Indianapolis, IN, USA). To ensure sample collection, the GCF volume was determined based on measurements of weighing the tubes and strips before and after sampling using a very sensitive scale^26^ (readability of 0.01 mg; Explorer Semi Micro Ex125M, OHAUS, Greifensee, Switzerland). All the samples collected had volumes of GCF ≥ 10 µl. After obtaining the supernatant, the GCF samples were frozen at −80 °C until further biochemical analysis.

### Quantification of cytokines in gingival crevicular fluid using multiplexed bead immunoassays

GCF cytokine levels were determined using the human cytokine 16-plex Procarta immunoassay (Affymetrix, Inc., Santa Clara, CA, USA). Sixteen mediators were measured: granulocyte-macrophage colony stimulating factor – GMCSF; IFNgamma; IL1alpha; IL1beta; IL2; IL3; IL4; IL5; IL6; IL10; IL12p40; IL12p70; IL13; IL17A; IL17F; and TNFalpha.

A single investigator blinded to the clinical data performed the experimental analyses of the GCF cytokine quantification. The assays were performed in 96-well filter plates following the manufacturer’s instructions and applying an analysis protocol described previously^25^. The GCF samples were quantified using the Luminex 100™ instrument (Luminex Corporation, Austin, Texas, USA) and all of them were run in duplicate. The concentrations of the unknown samples were estimated from the standard curve using a 5PL algorithm and the Luminex IS 2.3 and xPONENT 3.1 software packages (Luminex Software, Inc.). Values were expressed as pg/ml adjusting for the dilution factor. Samples below the detection limit (DL) of the assay were recorded as DL/2^27^, while those above the upper limit of quantification of the standard curves were assigned the highest value of the curve.

### Statistical analysis

The statistical analyses were performed using the R software (version 3.4.3)^28^. It is available as Free Software under the terms of the Free Software Foundation’s GNU General Public License in source code form. After applying the Shapiro-Wilks test and verifying the non-normal distribution of almost all the clinical variables, the Mann-Whitney U test was used to compare the quantitative variables between the perio and control groups. The Fisher’s exact test was used to assess the association of the qualitative variables between both study groups. The significance level applied was a *p-*value < 0.05.

### Comparison of GCF cytokine levels and cytokine ratio values in periodontally healthy individuals and patients with chronic periodontitis

**Figure 1:**
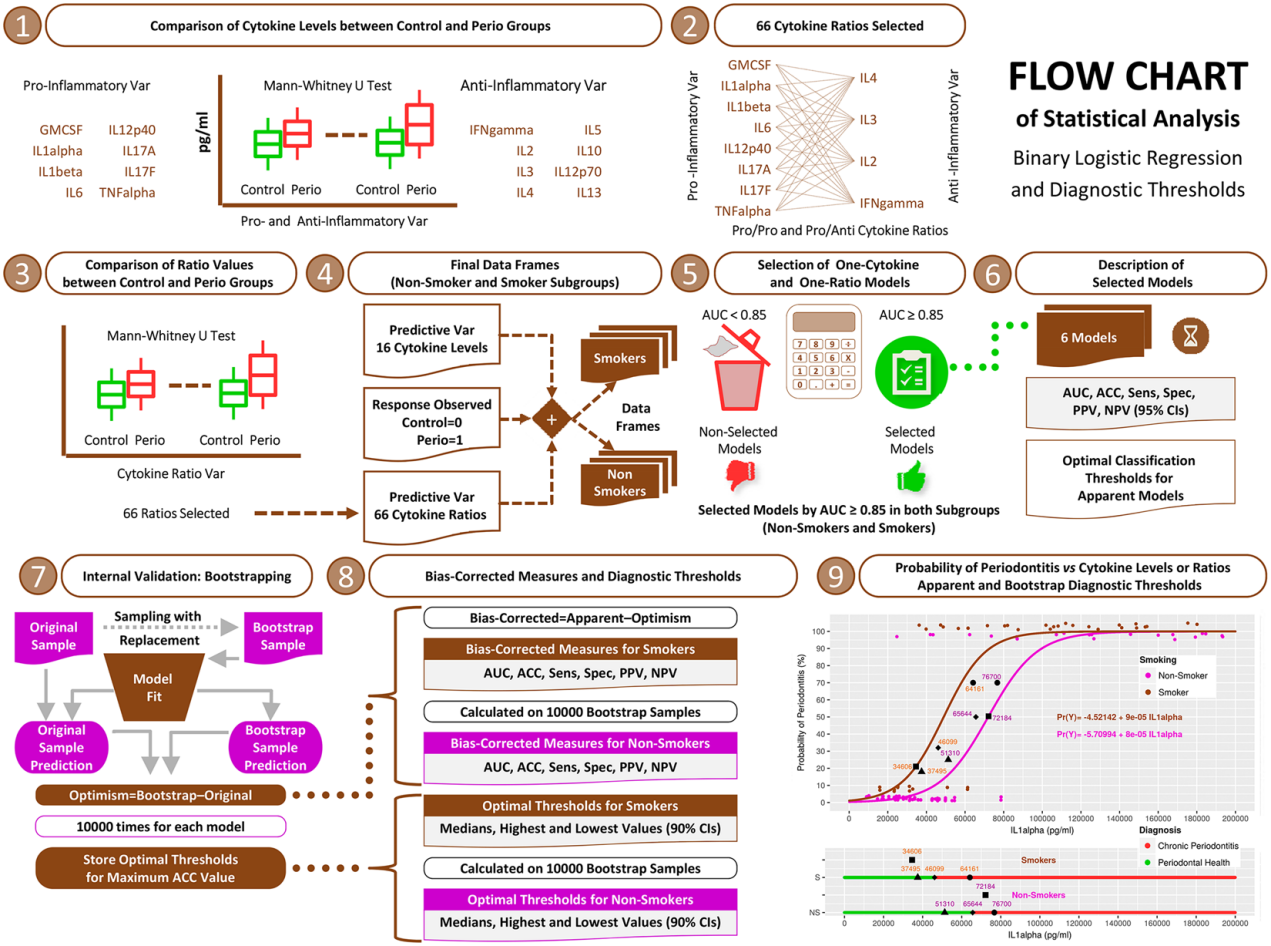
Flow chart of the statistical analysis: binary logistic regression and diagnostic thresholds. AUC: area under the curve; ACC: accuracy; Sens: sensitivity; Spec: specificity; PPV: positive predictive value; NPV: negative predictive value; CIs: confidence intervals.

After verifying the non-normal distribution of variables using the Shapiro-Wilks test, the Mann-Whitney U test was used to compare the cytokine levels and cytokine ratios in the control and perio groups. The significance levels applied were adjusted by the Benjamini-Hochberg correction^29^, with *p*-values ≤ 1 × 10^−3^ and <1 × 10^−5^, respectively. A total of 66 cytokine ratios were evaluated, taking into account exclusively those cytokines that showed significant levels in the periodontal patients compared to the controls (Fig. 1).

### Predictive modelling of chronic periodontitis based on cytokine levels and cytokine ratios: model selection; discrimination and classification measures; determination of diagnostic thresholds; and internal validation

To obtain specific diagnostic thresholds differentiating by smoking status, we decided to develop different models for non-smokers and smokers (n = 93 and 54, respectively). Models were constructed by selecting one cytokine or cytokine ratio as a predictor variable, which was treated in its original scale.

The statistical criterion applied for the model selection was the ability of each cytokine- or cytokine ratio-based model to determine the presence of chronic periodontitis using the value of the area under the curve (AUC)^30^. The AUC values and their corresponding 95% confidence intervals (CIs) obtained by bootstrapping were calculated using the pROC package (version 1.10.0)^31^. Only those models that presented an apparent AUC ≥ 0.85 in both types of model for smokers and non-smokers were selected^32^.

The best cut-off value or optimal classification threshold for each model was defined as that which provides the maximum percentage of correct predictions (accuracy, ACC), and was calculated using the PresenceAbsence package (version 1.1.9)^33^. By setting this optimal value, various classification measures such as the ACC, sensitivity, specificity, positive predictive value (PPV), and negative predictive value (NPV), as well as their corresponding 95% CIs acquired by bootstrapping, were obtained using the pROC package^31^. The respective cytokine levels or cytokine ratios were calculated for all the periodontitis probability values of each model, and the model curves were constructed graphically using the ggplot package (version 2.2.1)^34^.

Regarding internal validation, bootstrapping was used to test for possible overfitting by determining the optimism values on the discrimination and classification measures. The bootstrap analysis was replicated on 10,000 random samples of the same sample size, drawn with replacements from the original sample^35,36^. Bias-corrected (bc) AUC and all other classification measures (bc-sensitivity, bc-specificity, bc-PPV, bc-NPV) were calculated as their corresponding apparent measures derived from the entire original sample minus optimism^35,36^. This technique was also used to define the cytokine thresholds for the median ACC values derived from 10,000 samples from each model selected, as well as the thresholds for the 90% CIs of the ACC values (Fig. 1).

### Ethics approval and consent to participate

The study’s protocol was approved by the Clinical Research Ethics Committee of Galicia (number 2015/006). Patients who agreed to participate in the research provided written informed consent.

## Results

The mean age of the study group was 48.37 ± 11.55 years; 62 individuals were male and 85 female. The perio group had significantly higher BPL, BOP, PPD and CAL values than the control group at both the full mouth and sampling site levels (*p* < 0.001; Table 1). The number of smokers was significantly higher in the perio group than in the control group (41 and 13 patients, respectively, *p* < 0.001; Table 1).

### Comparison of GCF cytokine levels and cytokine ratio values in periodontally healthy individuals and patients with chronic periodontitis

All the pro-inflammatory cytokines analysed (GMCSF, IL1alpha, IL1beta, IL6, IL12p40, IL17A, IL17F and TNFalpha), as well as four cytokines with anti-inflammatory effects (IFNgamma, IL2, IL3 and IL4), had significantly higher levels in the perio group than in the control group (adjusted *p*-value ≤ 1 × 10^−3^). Nineteen cytokine ratios showed significant differences between the control and perio groups (adjusted *p*-value < 1 × 10^−5^). Of these ratios, nine were based on the combination of two pro-inflammatory cytokines, which were: IL1alpha combined with GMCSF, IL12p40 and TNFalpha; and IL1beta combined with GMCSF, IL12p40, IL17F or TNFalpha, GMCSF/IL17A and IL17A/IL17F. The remaining 10 ratios were based on the combination of one pro-inflammatory cytokine and one cytokine with anti-inflammatory effects. These were: IL1alpha/IL2; IL1alpha/IL3; IL1alpha/IFNgamma; ILbeta/IL2; ILbeta/IL3; ILbeta/IL4; ILbeta/IFNgamma; IL17A/IL2; IL17A/IL3; and IL17A/IFNgamma. All these cytokine ratios, except for GMCSF/IL17A, had significantly higher values in the perio group than in the control group (Table 2).

**Table 2:** Concentrations of cytokines and cytokine ratios that showed significant differences (adjusted *p*-values ≤ 1 × 10^−3^ and <1 × 10^−5^, respectively) between the control and perio groups.

CGF Cytokine Concentration (pg/ml)	Median (IQR)		
	Control group	Perio group	Adjusted p-value
GMCSF	150.24 (129.67)	247.24 (255.55)	7.29E-05
IL1alpha	30405.78 (20713.06)	148825.83 (221175.10)	2.64E-21
IL1beta	2881.75 (1958.43)	17947.50 (17308.08)	5.59E-21
IL6	166.71 (163.90)	313.45 (461.34)	3.77E-06
IL12p40	7.34 (5.65)	17.30 (11.06)	7.10E-12
IL17A	7.53 (9.38)	28.45 (25.83)	9.70E-19
IL17F	3.40 (7.01)	9.62 (15.47)	7.75E-09
TNFalpha	6.46 (13.49)	22.75 (15.95)	2.50E-04
IFNgamma	4.71 (3.85)	9.55 (9.60)	3.42E-07
IL2	9.96 (8.74)	14.96 (8.08)	0.001
IL3	54.26 (37.15)	99.60 (89.75)	1.23E-05
IL4	5.96 (21.42)	12.37 (47.02)	2.50E-04
**CGF Cytokine Ratio**			
GMCSF/IL17A	26.57 (25.02)	7.76 (14.82)	2.80E-08
IL1alpha/GMCSF	218.44 (198.78)	631.24 (1434.02)	1.03E-11
IL1alpha/IL12p40	4531.12 (3831.16)	6849.23 (17879.96)	2.78E-06
IL1alpha/TNFalpha	4524.95 (5930.96)	8427.24 (22799.21)	7.71E-06
IL1beta/GMCSF	19.52 (24.42)	67.75 (97.45)	1.18E-12
IL1beta/IL12p40	402.43 (516.02)	844.61 (1378.24)	2.83E-08
IL1beta/IL17F	662.10 (964.59)	1446.37 (3287.34)	1.62E-06
IL1beta/TNFalpha	468.80 (583.89)	854.64 (1669.85)	2.49E-07
IL17A/IL17F	0.88 (2.46)	2.31 (4.79)	1.88E-06
IL1alpha/IL2	3279.43 (3601.17)	8890.94 (17774.01)	1.93E-14
IL1alpha/IL3	630.99 (759.95)	2262.85 (5590.86)	1.42E-10
IL1alpha/IFNgamma	7728.27 (4744.27)	18426.38 (52652.97)	3.05E-12
IL1beta/IL2	260.08 (210.23)	1249.52 (1480.05)	4.16E-14
IL1beta/IL3	66.56 (87.48)	206.71 (469.16)	8.04E-11
IL1beta/IL4	536.43 (702.25)	1530.57 (2756.95)	7.25E-06
IL1beta/IFNgamma	805.90 (387.53)	2084.77 (5776.57)	5.54E-13
IL17A/IL2	0.64 (0.58)	1.96 (1.41)	5.54E-13
IL17A/IL3	0.13 (0.12)	0.34 (0.32)	4.80E-11
IL17A/IFNgamma	1.81 (1.03)	3.14 (2.03)	4.20E-12

IQR, interquartile range; CGF, crevicular gingival fluid. Concentration range for each biomarker analysed: GMCSF, 0.53–55,050 pg/ml; IFNgamma, 0.02–6,650 pg/ml; IL1alpha, 0.34–28,800 pg/ml; IL1beta, 0.09–23,150 pg/ml; IL2, 0.04–13,700 pg/ml; IL3, 0.19–26,500 pg/ml; IL4, 0.10–29,250 pg/ml; IL5, 0.04–17,800 pg/ml; IL6, 0.10–27,200 pg/ml; IL10, 0.04–10,050 pg/ml; IL12p40, 0.14–27,350 pg/ml; IL12p70, 0.26–18,050 pg/ml; IL13, 0.34–23,700 pg/ml; IL17A, 0.36–30,900 pg/ml; IL17F, 0.25–34,700 pg/ml; TNFalpha, 0.21–16,800 pg/ml.

### Predictive modelling of chronic periodontitis based on GCF cytokine levels and cytokine ratios: model selection; discrimination and classification measures; determination of diagnostic thresholds; and internal validation

There were three cytokine-based models and three cytokine ratio-based models, which had an apparent AUC ≥ 0.85 for both non-smokers and smokers. These models were IL1alpha, IL1beta, IL17A, IL1alpha/IL2, IL1beta/IL2 and IL17A/IL2.

Apparent and bc-percentages of discrimination and classification of the six predictive models are described in Table 3. The cytokine-based models had AUC and bc-AUC values ≥ 0.940 and ≥ 0.912, respectively, and the cytokine ratio-based model values were ≥ 0.857 and ≥ 0.834, respectively. The bc-ACC range derived from the cytokine-based models was 86.8–94.1% and that of the cytokine ratio-based models was 72.9–88.7%, with IL17A and IL17A/IL2 being the biomarkers with the lowest bc-ACC values in both smokers and non-smokers. The 95% CIs of the model coefficients and those of the performance measures are detailed in Supplementary Dataset 1.

**Table 3:** Apparent and bias-corrected measures of discrimination and classification of the models based on cytokines and citokine ratios for both smokers and non-smokers.

Model	Smoking status	AUC	ACC (%)	Sensitivity (%)	Specificity (%)	Positive Predictive Value (%)	Negative Predictive Value (%)
IL1alpha	Smoker	0.966 0.951	92.5 89.4	100.0 97.1	69.2 65.8	91.1 89.9	100.0 92.6
	Non-smoker	0.959 0.958	93.5 92.4	87.5 85.8	96.7 96.0	93.5 92.1	93.7 92.8
IL1beta	Smoker	0.968 0.945	94.4 90.7	97.5 96.6	84.6 71.1	95.2 91.9	92.3 88.8
	Non-smoker	0.944 0.943	94.6 94.1	90.6 89.5	96.7 96.5	93.7 93.4	95.2 94.6
IL17A	Smoker	0.940 0.912	92.5 90.3	95.1 93.9	84.6 79.1	95.2 93.6	85.7 82.1
	Non-smoker	0.914 0.914	88.1 86.8	78.1 76.1	93.4 92.5	86.2 84.3	89.2 88.2
IL1alpha/IL2	Smoker	0.878 0.868	85.1 81.1	100.0 99.0	38.4 29.5	83.6 80.2	100.0 99.0
	Non-smoker	0.911 0.905	88.1 85.1	84.3 80.0	90.1 88.6	81.8 78.6	91.6 89.2
IL1beta/IL2	Smoker	0.906 0.896	92.5 88.7	95.1 94.7	84.6 76.3	95.1 91.1	85.7 82.1
	Non-smoker	0.886 0.881	84.9 79.5	81.2 70.6	86.8 84.3	76.4 72.3	89.8 84.1
IL17A/L2	Smoker	0.955 0.948	92.5 87.2	100.0 98.6	69.2 51.4	91.1 86.5	100.0 95.7
	Non-smoker	0.857 0.834	80.6 72.9	87.5 81.7	77.0 68.3	66.6 54.3	92.3 89.1

In each cell, the first value is referred to the apparent performance measures and the second to the corrected performance measures by the level of optimism, calculated using a bootstrap procedure. The 95% CIs of the different performances measures are detailed in Supplementary Dataset 1.

**Figure 2:**
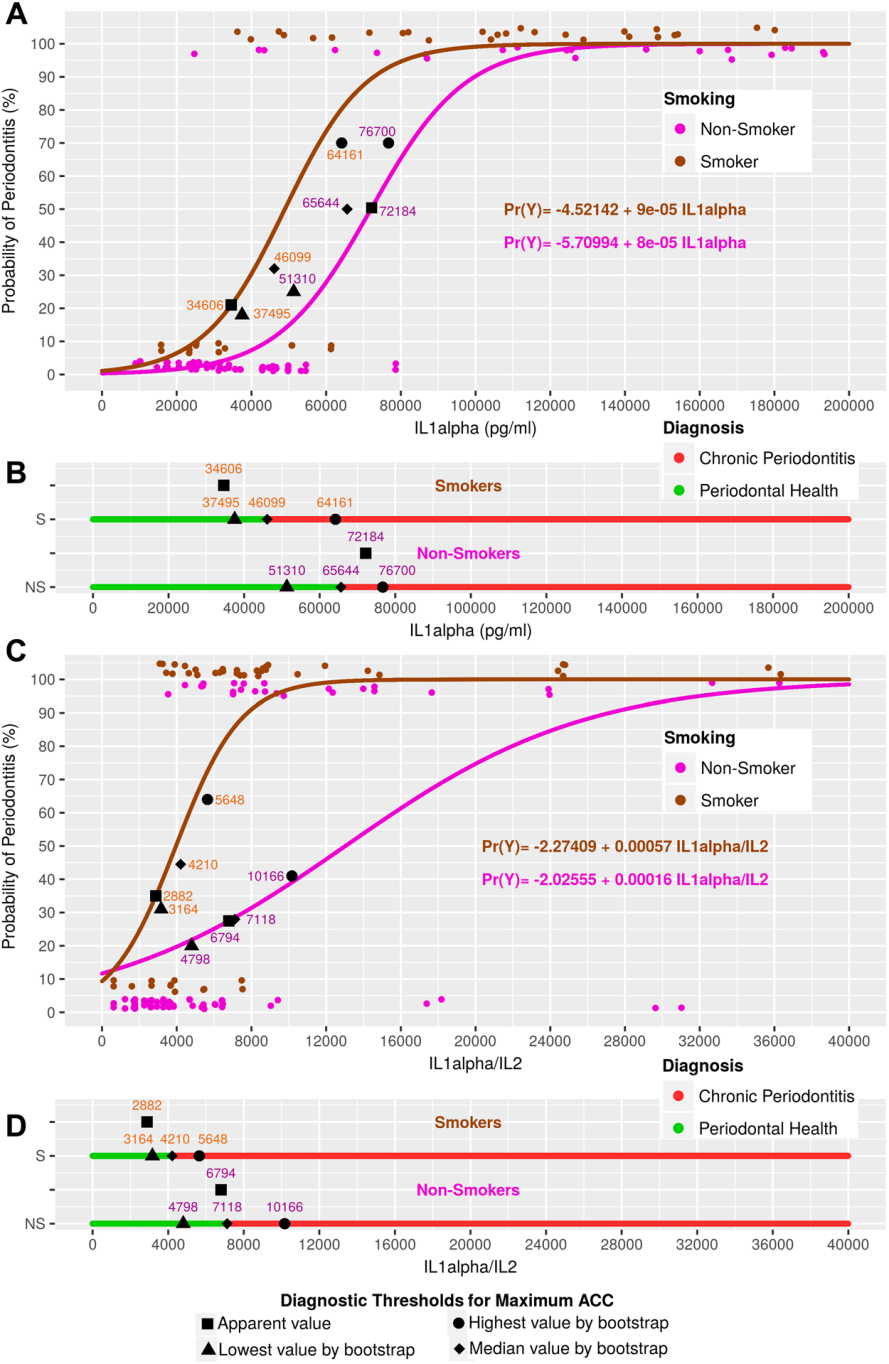
Model curves based on IL1alpha and IL1alpha/IL2, defining the diagnostic thresholds for apparent and median ACC values, as well as those thresholds for the 90% CIs of the ACC values.

**Figure 3:**
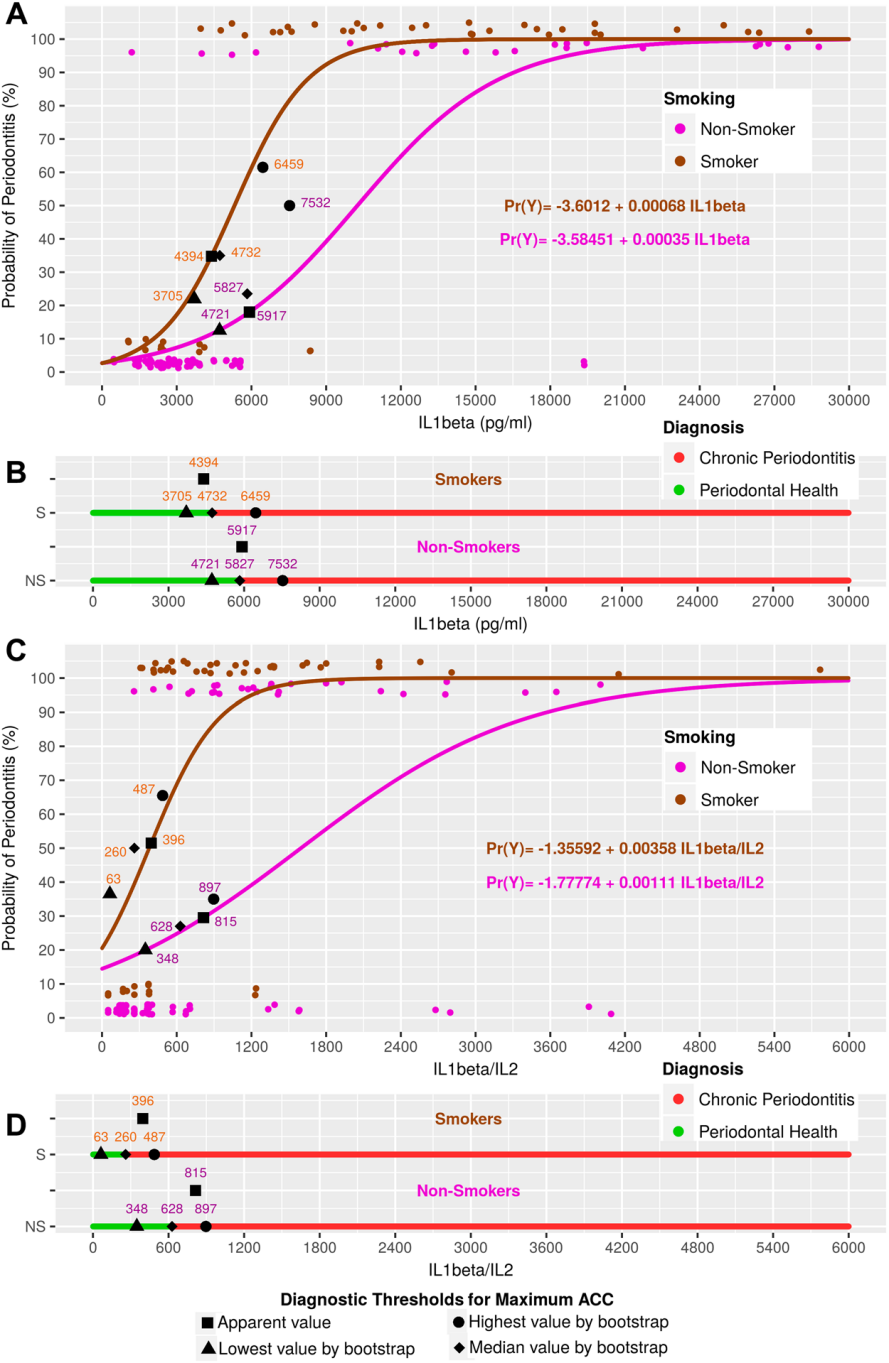
Model curves based on IL1beta and IL1beta/IL2, defining the diagnostic thresholds for apparent and median ACC values, as well as those thresholds for the 90% CIs of the ACC values.

**Figure 4:**
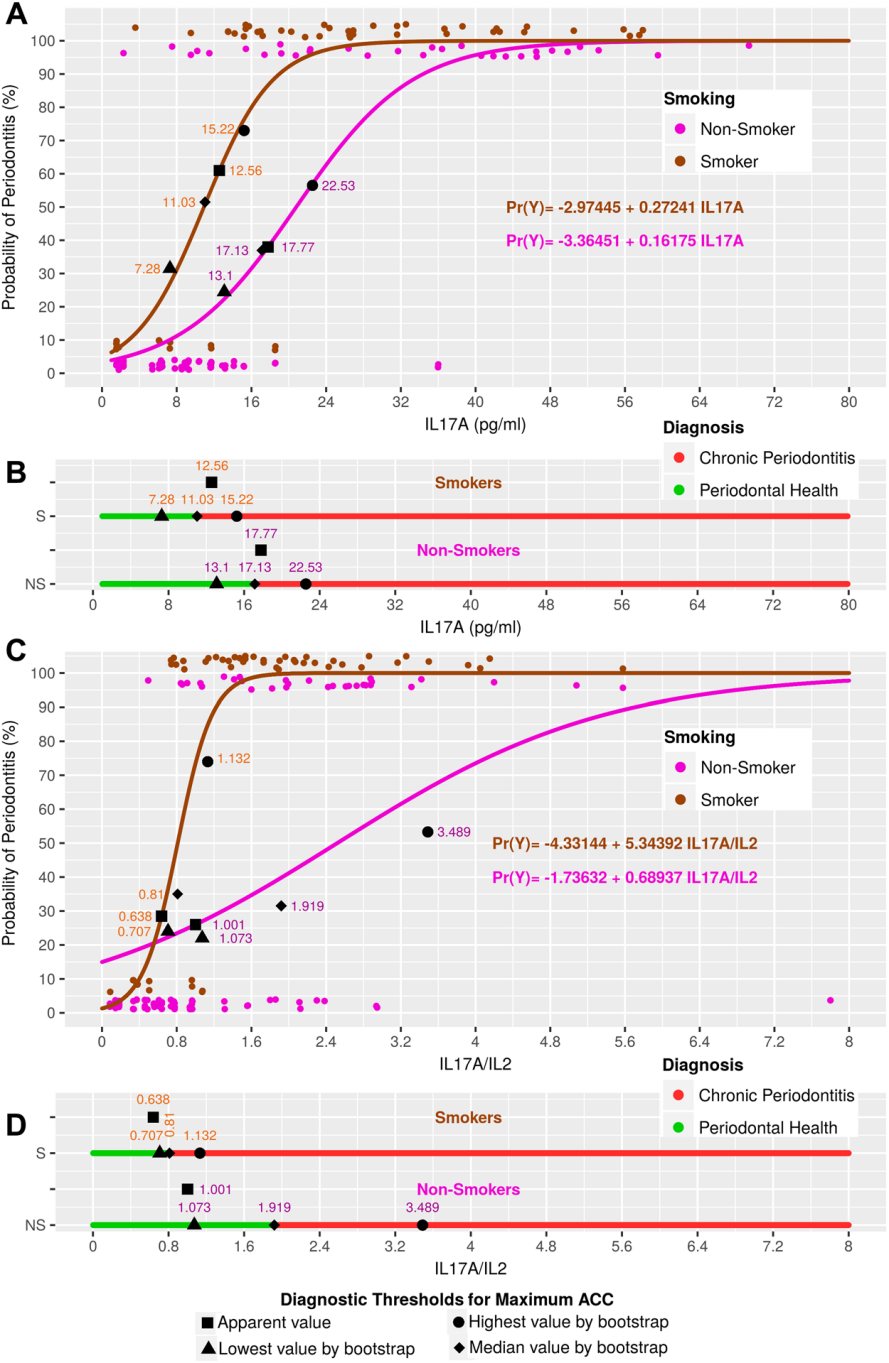
Model curves based on IL17A and IL17A/IL2, defining the diagnostic thresholds for apparent and median ACC values, as well as those thresholds for 90% CIs of the ACC values.

The periodontitis probability range for the median ACC values varied between 23 and 51%. The cytokine thresholds in pg/ml for the median ACC values (and those for the 95% CIs of the ACC values) for smokers and non-smokers were, respectively: IL1alpha model: 46099 (37495–64161) and 65644 (51310–76700); IL1beta model: 4732 (3705–6459) and 5827 (4721–7532); IL17A model: 11.03 (7.28–15.22) and 17.13 (13.10–22.53); IL1alpha/IL2 model: 4210 (3164–5648) and 7118 (4798–10166); IL1beta/IL2 model: 260 (63–487) and 628 (348–897); and IL17A/IL2 model: 0.810 (0.707–1.132) and 1.919 (1.073–3.489). The range of cytokine thresholds represented around 9–13% of the cytokine or ratio measurement range, except for IL17/IL2 for non-smokers (30%). Compared to the non-smokers, the smokers had lower diagnostic thresholds on all the predictive models for both apparent ACC values and ACC values obtained by bootstrapping (Figs 2–4).

## Discussion

### High cytokine concentrations and cytokine ratios in the gingival crevicular fluid of patients with chronic periodontitis

As mentioned in the Introduction, there has been a failure to study a broader spectrum of cytokines that may directly influence the local inflammatory response in different types of periodontitis^37^. The present series is the first comparative analysis of more than 50 cytokine ratios derived from the simultaneous quantification of 16 cytokines with different roles in the pathophysiology of chronic periodontitis^7,8^.

It should be noted that a particularly strict corrected significance value was applied (adjusted *p*-value < 1 × 10^−5^) in order to select the cytokine ratios with the most significant impact on chronic periodontitis. This statistical decision conditioned the ratios considered to be non-significant and significant. As a consequence, comparisons with the contributions of other authors must be interpreted with caution.

Although very few authors have investigated the ratios between pro-inflammatory cytokines in periodontitis^38–40^, up to eight pro-inflammatory cytokine ratios showed significant differences in periodontal patients. Although we detected significantly elevated levels of all the pro-inflammatory cytokines analysed, IL1alpha and IL1beta were the most important biomarkers in terms of increased concentration associated with the disease. This resulted that the ratios based on IL1alpha combined with GMCSF, IL12p40 or TNFalpha, and IL1beta combined with GMCSF, IL12p40, IL17F or TNFalpha, showed significantly higher values in the periodontal patients. Coinciding with the results reported by Azman *et al*.^41^, we also obtained a significantly elevated IL17A/IL17F ratio in the patients with chronic periodontitis. Interestingly, in this series, and unlike the other pro-inflammatory cytokine ratios, the GMCSF/IL17A ratio had significantly lower values in the periodontal patients, representing the first evidence of the impact of this ratio in the pathogenesis of periodontitis.

Most previous studies have focused on the analysis of ratios between pro-inflammatory and anti-inflammatory cytokines, or vice versa, in periodontal diseases, with IL1beta/IL10 and IL11/IL17 being the most evaluated^14,38,42–45^. In the present series, up to nine ratios based on the combination of one pro-inflammatory cytokine (IL1alpha, ILbeta or IL17A) and one cytokine with anti-inflammatory effects (IFNgamma, IL2, IL3 or IL4) showed significantly higher values in the periodontal patients. These results were due to the higher mean concentrations of pro-inflammatory cytokines compared to the levels presented by anti-inflammatory cytokines. In contrast to the findings of Stadler *et al*.^46^, these mediators also showed a significant mean increase associated with chronic periodontitis. On the other hand, applying multivariate predictive modelling techniques, we have previously demonstrated that the extent of the periodontitis-associated imbalance between IL1alpha, ILbeta or IL17A (acting as enhancers) and IFNgamma, IL2, IL3 or IL4 (acting as protectors) was associated with a particular probability of having chronic periodontitis^25^.

We have not found any articles that would enable us to compare our findings on ratios between IL1alpha and different anti-inflammatory cytokines. Regarding the ratios between ILbeta and other anti-inflammatory cytokines, some authors have observed that: the ILbeta/IL10 ratio was increased in the GCF or gingival tissue of patients with aggressive periodontitis or chronic periodontitis^14,42,43^; this ratio was significantly reduced after periodontal therapy^43^. However, after studying these papers in detail, these results can be attributed mainly to significantly higher mean levels of IL1beta, while the levels of IL10 showed non-significant individual variations. These results obtained *in vivo* call into question the importance of this ratio in the pathogenesis of periodontitis. Likewise, in the present study, no significant differences in IL10 levels between the controls and periodontal patients were detected, and so the IL1beta/IL10 ratio was not evaluated. However, it should be noted that IL10 acquired a greater protagonism as an anti-inflammatory cytokine within a two-biomarker predictive model, as this increased the capacity of IL1beta to discriminate the chronic periodontitis state^25^. In contrast, in the present series, we observed that other ratios, such as IL1beta/IFNgamma, ILbeta/IL2, ILbeta/IL3 and ILbeta/IL4, may play an essential role in quantitative terms in chronic periodontitis. Several studies have revealed that the IL11/IL17 ratio was reduced in patients with chronic and aggressive periodontitis^44,45,47^, although other authors have described conflicting findings^38^. In the present study, other ratios such as IL17A/IFNgamma, IL17A/IL2, IL17A/IL3 and IL17A/IL4 had significantly higher values in the periodontal patients, reflecting their impact on chronic periodontitis.

Consequently, this study is the first time that evidence is provided on a high number of ratios between pro-inflammatory cytokines or pro-inflammatory and anti-inflammatory cytokines that, due to their performance in GCF samples, could be biomarkers associated with chronic periodontitis. Future research is required to clarify the relevance of these ratios in the chronic periodontitis pathogenesis.

### High predictive ability of GCF cytokine levels and cytokine ratios for the diagnosis of chronic periodontitis

Due to the characteristics of cytokine networks^48^, whether cytokines in GCF may show an acceptable ability to discriminate chronic periodontitis from periodontal health is questioned. However, this affirmation is supported by little evidence, as there are very few studies that have evaluated the predictive properties of cytokines in chronic and aggressive periodontitis using an appropriate experimental design^19,20^. The current series reveals the first results on the predictive ability of cytokines and cytokine ratios for the diagnosis of chronic periodontitis, differentiating between smokers and non-smokers. Moreover, internal validation was carried out for the first time on the predictive parameters obtained, as recommended in the TRIPOD guidelines^24^.

In this study, in relation to individual cytokines, and corroborating observations published previously by our research group^25^, there were three models consisting of IL1alpha, IL1beta and IL17A, which presented a bc-AUC > 0.90 for both smokers and non-smokers. According to experts in the field^32^, these high AUC values indicate that these pro-inflammatory cytokines have a great capacity to discriminate the disease condition. Consequently, these pro-inflammatory cytokines were associated with elevated bc-ACC percentages: 90.7% (for IL1beta), 90.3% (for IL17A) and 89.4% (for IL1alpha) in smokers; and 94.1%, 86.8% and 92.4%, respectively, in non-smokers. Findings on IL1’s high predictive ability are consistent with those previously described by Baeza *et al*.^20^, while IL17’s findings represent the first evidence of a strong diagnostic capability. In our opinion, our results on the high predictive potential of these cytokines are comparable to those found for other well-known biomarkers, such as different metalloproteinases^20^.

We evaluated the cytokine ratios using predictive modelling techniques, with the aim being to identify a set of biomarkers that guarantee a high diagnostic predictability^16^. In this sense, we obtained three ratio-based models consisting of IL1alpha/IL2, IL1beta/IL2 and IL17A/IL2, which presented a bc-AUC > 0.80 for both smokers and non-smokers. These bc-AUC values, although lower than those detected in individual cytokines, were also very high, revealing that these cytokine ratios were associated with an excellent ability to discriminate periodontitis patients^32^.

For the first time in the literature, we have defined specific thresholds with diagnostic potential for each smoking status. These were derived from cytokine- and cytokine ratio-based predictive models, and their validity was verified given that the apparent ACC and median ACC values derived from the bootstrap approaches were similar. On the other hand, the range of thresholds obtained by bootstrapping represented only around 9–13% of the measurement range of the biomarkers (except for the IL17A/IL2 ratio in non-smokers). Accordingly, the upper and lower thresholds of these ranges would ensure optimal diagnostic classification.

In line with the trend of attempting to discover biomarkers to improve the clinical diagnosis of periodontal diseases^16–18^, the determination of these specific thresholds could represent a first step in the design and construction of chronic periodontitis diagnostic kits for use in clinical practice.

As smoking is a well-established traditional risk factor for chronic periodontitis^49,50^, we demonstrated previously from a predictive perspective that smoking status increases the probability of having chronic periodontitis by 15–20%^25^. Interestingly, in the present series, smokers had lower diagnostic thresholds than non-smokers. At a biochemical level, this justifies what is observed at a clinical level, i.e. the presence of a less intense inflammatory reaction in smoking-associated periodontitis, indicating that smoking may have an immunosuppressant effect^49^. Secondly, it reveals the convenience of designing biomarker studies for predicting periodontal diseases differentiating by smoking status, especially if the diagnostic thresholds are to be defined.

Our research has some limitations. Although we are in a scenario of small data, the sample size used allowed certain metrics of model performance were estimated with an acceptable precision^24^; in addition, a strict model selection criterion (apparent AUC value ≥ 0.85) was applied for both non-smokers and smokers. An internal validation process was carried out using bootstrap techniques, with the aim being to counteract the prediction that the study’s accuracy is only measured in the samples that generated the model equations^24^. Although the results derived from the internal validation were quite optimal, the predictive parameters and diagnostic thresholds obtained from our models should be evaluated in an external cohort of patients (including using calibration analyses) to verify whether our findings are applicable universally.

In conclusion, a high number of previously undescribed GCF cytokine ratios are elevated in patients with chronic periodontitis, evidencing disease-associated imbalances between cytokines with pro-inflammatory and anti-inflammatory effects. IL1alpha, IL1beta and IL17A, and their ratios with IL2, are excellent diagnostic biomarkers in GCF for distinguishing periodontitis patients from periodontally healthy individuals. Cytokine thresholds in GCF with diagnostic potential are defined, showing that smokers have lower threshold values than non-smokers.

## Electronic supplementary material

Dataset 1

## Data Availability

All results obtained in this study are included in this manuscript and in the supplementary information file.
